# Nitrogen addition alters soil microbial community structure and decrease nitrogen cycling gene abundance in acidic macadamia orchard soils

**DOI:** 10.3389/fmicb.2026.1878175

**Published:** 2026-10-08

**Authors:** Linyu Qu, Tinghong Ye, Xiyong He, Liping Yang, Xiaorong Huang, Zhe Chen, Haiqing Zhang

**Affiliations:** 1 Yunnan Institute of Tropical Crops, Jinghong, Yunnan, China; 2 Yunnan Agricultural Engineering Research Center for Macadamia, Jinghong, Yunnan, China; 3 Yunnan Technology Innovation Center of Woody Oil, Jinghong, Yunnan, China

**Keywords:** macadamia, microbial community, network, nitrogen addition, nitrogen cycling

## Abstract

Nitrogen addition can alter soil microbial communities and regulate nitrogen cycling processes. However, the role of nitrogen-induced soil acidification in modulating nitrogen cycling in macadamia orchard soils remains poorly understood. Based on a 3-year field fertilization experiment, this study established three treatments: no-nitrogen (N0), medium-nitrogen (N1), and high-nitrogen (N2). We systematically investigate the effects of nitrogen addition on soil chemical properties, the abundance of nitrogen cycling genes, enzyme activities, microbial community structure, and co-occurrence networks. The results showed that nitrogen addition significantly increased the contents of alkaline hydrolyzable nitrogen (AHN) and nitrate nitrogen (NO_3_^–^-N), altered soil stoichiometric ratios, and exacerbated soil acidification. Quantitative PCR (qPCR) analysis revealed that nitrogen addition significantly decreased the abundance of nitrification genes (AOA-*amoA*), ammonification gene (*ureC*), and denitrification genes (*narG*, *nirK*, *nosZ*), while no significant changes were observed for AOB-*amoA* or *nirS*. 16S rRNA gene sequencing demonstrated that nitrogen addition altered the succession of microbial communities towards a copiotrophic (R-strategy) state and altered the predicted carbon-metabolism functional profiles. Co-occurrence network analysis showed that the N2 treatment reduced network complexity but enhanced modularity, suggesting that microbial communities may respond to nitrogen addition through functional modular reorganization. The asynchronous changes between key enzyme activities and their corresponding gene abundances revealed the complex regulatory mechanisms of the nitrogen cycle. Furthermore, correlation analyses identified pH, electrical conductivity (EC), and NO_3_^–^-N were important environmental factors affecting nitrogen cycle function gene groups. These findings provide new insights into the response mechanisms of the nitrogen cycle in the soil ecosystem of Macadamia orchards.

## 1 Introduction

With the advancement of intensive agricultural production, increased fossil fuel combustion, and intensified artificial fertilization activities, external nitrogen additions have become a major driving force altering the global soil ecological environment (Fowler et al., 2013; Zhu et al., 2025). Macadamia (*Macadamia* spp.), a member of the Proteaceae family, is native to southeastern Queensland and northeastern New South Wales, Australia (Zhao et al., 2019; Mai et al., 2020). As a commercial fruit widely cultivated in tropical and subtropical regions around the world, macadamia is extensively grown in countries such as Australia, South Africa, Kenya, China and the United States (Global Info Research, 2025),with China being the largest producer (Zhang et al., 2026). Nitrogen, a key nutrient limiting plant growth and yield, is an important measure for achieving high-yield and high-quality production in macadamia cultivation (Gallart et al., 2021; Tao et al., 2022). However, excessive or improper nitrogen additions not only affect crop growth but also pose potential threats to the sustainability of soil ecosystems.

Soil microorganisms, as key drivers of nitrogen cycling, exhibit high sensitivity to exogenous nitrogen. Extensive research indicates that exogenous nitrogen addition disrupts the original stoichiometric balance of soil, as well as the physicochemical properties’ steady state, directly or indirectly regulating microbial growth, reproduction, and metabolic processes (Geisseler and Scow, 2014; Cui et al., 2024; Du et al., 2024). Specifically, extensive research has confirmed that in temperate cereal crop systems such as wheat and corn, nitrogen addition not only induces soil acidification, exacerbating nutrient imbalance and disrupting microbial metabolic equilibrium (Ullah et al., 2019; Li H. et al., 2025). Soil acidification is a prominent issue in Yunnan Province, where over 30% of cultivated soils have already declined to pH values below 5.5 (Li, 2022), and this acidification trend continues to intensify with under excessive nitrogen fertilization. Studies conducted in tropical and subtropical cropping systems of Yunnan Province have further confirmed that excessive nitrogen input is a major driver of soil acidification (Chen et al., 2024; Ye et al., 2025). Macadamia is a typical perennial woody cash crop in Yunnan, and the soil acidification trend in its orchards should not be overlooked.

Due to excessive nitrogen addition, the excessive accumulation of eutrophic microbial communities, the destruction of species composition ratio and the decline of bacterial diversity have been widely reported in various ecosystems (Wang C. et al., 2018; Hu et al., 2024; Zhao et al., 2025). These changes alter the structure of soil microbial communities. At the same time, soil microorganisms disrupt the dynamic balance of soil nitrogen transformation by regulating the abundance and of key functional genes involved in nitrogen cycling (Wang C. et al., 2018; Li et al., 2020), as well as the activity of key enzymes (Li H. et al., 2025; Ren et al., 2025). This will affect the ecological stability and nutrient use efficiency of the crop system (Yu et al., 2019). It is noteworthy that there remains a lack of systematic understanding regarding how nitrogen cycling responds to nitrogen addition in tropical and subtropical cropping systems, particularly within the specific ecosystem of macadamia plantations (Ma et al., 2023; Schaedel et al., 2023; Zhu et al., 2023). Although the effects of nitrogen fertilization on the soil physicochemical properties of tropical and subtropical macadamia orchards are preliminarily understood (Maselesele et al., 2021; Tan et al., 2024), the mechanisms by which the soil environment regulates microbially driven nitrogen cycling processes under different nitrogen addition rates remain unclear.

This study was based on a 3-year field experiment with a nitrogen fertilizer gradient, conducted in a subtropical macadamia orchard. Soil chemical properties, the abundance of nitrogen cycling genes, and enzyme activities were systematically analyzed using combined 16S rRNA gene sequencing and KEGG pathway analysis. The objective was to elucidate the response mechanisms of soil microbial community structure to nitrogen addition in acidic macadamia orchard soils, and to reveal the changes of key nitrogen cycling gene abundances and metabolic enzyme activities. These findings provide a theoretical basis for optimizing nitrogen addition strategies in macadamia orchards and for maintaining the stability of the soil ecosystem.

## 2 Materials and methods

### 2.1 Site description and sample collection

The research site is located in Xincheng Town, Yingjiang County, Dehong Dai and Jingpo Autonomous Prefecture, Yunnan Province, China (98.0774° E, 24.7817° N). The area has a subtropical monsoon climate, with an altitude of 1038 m, an average annual precipitation of 1464–1700 mm, and an average annual temperature of 19.3 °C. This study was conducted at a fixed-position nutrient observation trial site, using 10-year-old Own Choice (O.C.) cultivar trees as the experimental material. Three nitrogen addition treatments (N0, N1, N2) were established in this experiment, with three biological replicates for each treatment. Each replicate corresponded to an independent experimental plot containing six fruit trees. The trees were planted at a spacing of 5 m × 7 m, and each plot covered an area of 210 m^2^. All plots were arranged in a completely-randomized design in the field. Buffer zones of 10–14 m were set between adjacent experimental plots to prevent soil cross-contamination and inter-treatment interference, ensuring full spatial independence among all plots. 3-year continuous observation was carried out from October 2021 to October 2024. The experiment included three nitrogen addition treatments, each with nine replicates. Importantly, fertilization regimes were kept consistent over the entire 3-year experiment. Annual nitrogen application rates were 0 kg N tree^–1^ year^–1^ (N0), 0.95 kg N tree^–1^year^–1^ (N1), and 1.90 kg N tree^–1^year^–1^ (N2), equivalent to pure-nitrogen inputs of 0, 271.7 and 543.4 kg N ha^–1^year^–1^, respectively. Phosphorus (83 g tree^–1^ year^–1^) and potassium (1109 g tree^–1^ year^–1^) fertilizer doses were identical across all treatments. The fertilizers used in the experiment were urea (containing 46% N), calcium magnesium phosphate (containing 15% P_2_O_5_), and potassium sulfate (containing 52% K_2_O). Fertilizers were applied according to the following schedule: the post-fruit fertilizer (October, after fruit harvest) supplied 45% of the total nitrogen, 40% of the total potassium, and 100% of the total phosphorus; the flower fertilizer (February, during flower bud differentiation) supplied 15% of the total nitrogen and 10% of the total potassium; and the fruit-strengthening fertilizers were applied during the fruit expansion stage (May) and the oil accumulation stage (July), with each addition supplying 20% of the total nitrogen and 25% of the total potassium.

The soil samples were collected during the fruit harvest period in September 2024. A soil auger with a diameter of 5 cm was used to collect topsoil samples at a depth of 20 cm. Under the crown of each fruit tree, one sampling point was laid out respectively in the four orientations of east, south, west and north at a straight-line distance of 50–60 cm from the trunk, with four sampling points in total; all sampling sites were arranged to avoid fertilization ditches. Soil samples collected from two fruit trees within the same experimental plot were fully homogenized and combined into a single composite sample (approximately 1000 g of fresh soil). Visible gravel, plant roots, and residual plant debris were manually removed. The fresh composite soil was immediately divided into two portions. One portion was subpackaged, snap-frozen in liquid nitrogen, and stored at -80 °C for the determination of soil nitrate nitrogen (NO_3_^–^-N), ammonium nitrogen (NH_4_^–^-N), soil enzyme activity, qPCR quantification, and 16S rRNA gene sequencing. The remaining portion was air-dried in a cool shaded environment, ground, and sieved through a 2 mm mesh for general soil chemical analysis. Subsamples for soil organic matter (OM) measurement were further sieved through a 0.147 mm mesh.

### 2.2 Determination of soil chemical properties and soil enzyme activity

The soil pH and EC were measured using the international standard soil water ratio 1:5 method, and the pH and EC were measured using a SevenExcellence pH/Cond meter. (Mettler Toledo S470-k, Switzerland). The total nitrogen (TN) was determined by Automatic Kjeldahl nitrogen determinator (FOSS KJELTEC8400, Denmark). The content of soil OM was measured by potassium dichromate titration; AHN was measured by the alkali-hydrolysis diffusion method. The contents of available phosphorus (AP), NO_3_^–^N and NH4^++^-N were determined by flow injection analysis. Available potassium (AK) was determined by flame atomic absorption spectrophotometer (AA-6300c, Japan), and total carbon (TC) was determined by organic carbon analyzer (enviro TOC, Germany). The activities of ammonia monooxygenase (AMO, Cat. No. AD9354), nitrate reductase (NAR, Cat. No. AD9248), nitrite reductase (NIR, Cat. No. AD9238), and urease (UE, Cat. No. AD9246) were measured using commercial ELISA kits (Wuhan Adanti Biotechnology Co., Ltd.). Soil samples were stored at –80 °C and thawed only once to prevent repeated freeze-thaw cycles. In brief, 500 mg frozen soil was homogenized with 0.01 mol/L PBS (pH 7.2–7.4, 1:10, w/v), vortexed for 30 s, and centrifuged at 4000 rpm for 15 min to obtain the supernatant. The immune-binding reaction was incubated at 37 °C for 30 min. The immune-binding reaction was incubated at 37 °C for 30 min. Subsequent procedures followed the kit instructions. TMB chromogenic reagent was added, and color development proceeded for 15 min at 37 °C in the dark. Absorbance was read at 450 nm. Enzyme activity values were calculated from standard curves; samples were diluted 5-fold routinely, with additional dilution when OD exceeded the highest standard. The linear detection ranges were 1–45 U/L (AMO), 1.25–40 U/L (NAR), 2.5–80 U/L (NIR), and 30–1200 U/L (UE). Final enzyme activities were expressed as U/L in the supernatant.

### 2.3 Soil DNA extraction and gene quantitative PCR

Soil DNA was extracted using the OMEGA Soil DNA Kit (D5625-01, Omega Bio-tek, Norcross, GA, USA) following the manufacturer’s protocol. The extracted DNA was verified with 1% agarose gel. The following gene abundances were detected using qPCR: nitrification functional genes, i.e., the copy numbers of the *amoA* gene of ammonia-oxidizing archaea (AOA) and ammonia-oxidizing bacteria (AOB); denitrification genes *narG*, *nirS*, *nirK*, and *nosZ*; and the ammonification gene *ureC*. qPCR was performed with ArtiCanCEO SYBR qPCR Mix (Cat. No. TSE401, Tsingke) on a StepOne Plus system (ABI) in a total reaction volume of 20 μL, containing a final concentration of 0.4 μM for each forward and reverse primer and 1 μL of 5-fold-diluted DNA template; nuclease-free water was used to adjust the final volume. The thermal cycling program was as follows: 95°C for 2 min; 40 cycles of 95°C for 15 s, 60°C for 20 s, 72°C for 20 s. The melting-curve procedure was conducted as follows: 95 °C for 10 s, 65 °C for 1 min, followed by heating to 95°C at a ramp rate of 0.05 °C/s to confirm amplicon specificity. Standard curves were generated from 10-fold serial dilutions of plasmid standards constructed by TA cloning. At least five valid data points were retained after outlier removal, yielding *R*^2^ ≥ 0.999. The amplification efficiencies and limits of quantification (LOQ) of each target gene are listed in Table 1. Each sample was analyzed in triplicate technical replicates, and no-template negative controls were included in each qPCR run to detect potential contamination. Target gene copy numbers were calculated based on the standard curves and expressed as copies per gram of fresh soil.

**TABLE 1:** Primers used for quantitative PCR.

Gene name	Sequences (5′–3′)	Size (bp)	E (%)	LOQ (copies⋅μ L^−1^)	References
AOA-*amoA*	F:ATGGTCTGGCTWAGACG	491	88.6%	1.69 × 10^3^	Li et al., 2016
	R:GCCATCCATCTGTATGTCCA				
AOB *amoA*	F:GGGGTTTCTACTGGTGGT	483	93.6%	4.76 × 10^3^	He et al., 2021
	R:CCCCTCKGSAAAGCCTTCTTC				
*narG*	F:TAY GTS GGG CAG GAR AAA CTG	386	95.0%	6.08 × 10^3^	Li et al., 2016
	R:CGT AGA AGA AGC TGG TGG TGT T				
*nirS*	F:GTS AAC GTS AAG GAR ACS GG	601	85.6%	2.11 × 10^3^	Liao et al., 2018
	R:GAS TTC GGR TGS GTC TTG A				
*nirK*	F:ATCATGGTSCTGCCGCG	268	91.7%	4.69 × 10^3^	Li et al., 2016
	R:GCCTCGATCAGRTTGTGGTT				
*nosZ*	F:CGCRACGGCAASAAGGTSMSSGT	425	83.8%	2.45 × 10^3^	Liao et al., 2018
	R:CAKRTGCAKSGCRTGGCAGAA				
*ureC*	F:CGGCAAGGCCGGCAACCC	471	70.0%	5.05 × 10^3^	Oshiki et al., 2018
	R:GTBGHDCCCCARTCYTCRT				

### 2.4 16 S rRNA gene sequencing

Genomic DNA was extracted from samples, and its purity and integrity were assessed by agarose gel electrophoresis and Qubit fluorometry. The bacterial 16S rRNA V3-V4 region was amplified using primers 338F (5′-ACTCCTACGGGAGGCAGCAG-3′) and 806R (5′-GGACTACHVGGGTWTCTAAT-3′). The 20 μL PCR system contained 15 μL Phusion Master Mix, 0.2 μM of each primer, and 10 ng template DNA. Cycling conditions: 98 °C for 1 min; 30 cycles of 98 °C for 10 s, 50 °C for 30 s, 72 °C for 30 s; final extension at 72 °C for 5 min. Amplicons were bead-purified, quantified, pooled in equal amounts, and quality-checked using Qubit and qPCR before library construction. Sequencing (PE250) was performed on an Illumina NovaSeq 6000 platform by Novogene (Beijing, China) for all 27 soil samples. A total of 2,837,530 raw paired-end reads were obtained (74,028–104,886 reads per sample). Raw reads were demultiplexed and merged into 2,808,014 tags with FLASH v1.2.11. Quality filtering was carried out in fastp v0.23.1: ambiguous N-containing sequences were discarded, low-quality bases with a Phred score < 20 were trimmed, and merged tags shorter than 300 bp were removed, without a sliding-window filter. Chimeras were filtered using VSEARCH with reference-based alignment against the SILVA v138.1 database. Denoising into ASVs was implemented via the DADA2 plugin in QIIME2. Taxonomic assignment of representative ASVs was performed using a SILVA-138.1-trained naïve Bayes classifier; ASVs annotated as chloroplast or mitochondria were removed, with no extra abundance filtering. All samples were rarefied to a uniform depth of 65,224 reads. The Good’s coverage values across all samples ranged from 0.999 to 1.000.

### 2.5 Statistical analysis

One-way ANOVA followed by Tukey’s HSD post-hoc test was performed in GraphPad Prism v9.0.0 to evaluate nitrogen-addition effects on soil physicochemical properties, nitrogen cycling gene abundances, enzyme activities, bacterial α−diversity, and bacterial taxonomic relative abundances. qPCR gene abundance data were log_10_-transformed to satisfy parametric assumptions; residual normality and homogeneity of variances for these datasets were checked using the Shapiro-Wilk, Brown-Forsythe, and Bartlett’s tests. Bonferroni correction was applied for multiple pairwise comparisons, and the significance threshold was set at *P* < 0.05.

All multivariate analyses described below were conducted in R v4.5.2. Bacterial β−diversity was visualized by Bray-Curtis-based principal-coordinate analysis (PCoA). Permutational multivariate ANOVA (PERMANOVA, “adonis2” function in the vegan package, 999 permutations) was used to test overall community differences across treatments, and “betadisper” was applied to verify homogeneity of group dispersions. Redundancy analysis (RDA) was carried out to relate Z-score-standardized soil variables to bacterial community composition and functional gene abundances. Environmental variables with variance-inflation factor (VIF) > 10 were pre-removed to avoid multicollinearity, followed by forward variable selection. The global RDA model was evaluated with 9999 Monte Carlo permutations, and adjusted R^2^ values were reported. The significance of individual explanatory variables was assessed by permutation tests, with *p*-values adjusted using the Benjamini-Hochberg (BH) correction.

Mantel tests and Pearson correlation analyses were conducted to explore associations among soil properties, nitrogen cycling functional gene groups (ammonification, nitrification, and denitrification), and dominant bacterial phyla. Euclidean distances were computed for environmental variables, and Bray-Curtis distances were adopted for gene matrices; raw *p*-values were retained for Mantel tests. Pearson correlations were calculated without additional multiple-testing correction. Network topological parameters were calculated using the igraph package and visualized in Gephi. Differences in KEGG functional pathways among treatments were tested by one-way ANOVA implemented with the “aov” function in R v4.5.2. Since KEGG functional predictions were considered exploratory results requiring further experimental validation, no additional multiple-testing correction was performed for this dataset. Figures were plotted using the ggplot2 package in R v4.5.2.

## 3 Results

### 3.1 Soil chemical properties

Nitrogen addition significantly altered the soil chemistry of macadamia (Figure 1). With increasing nitrogen concentration, soil pH significantly decreased, whereas EC, carbon-to-nitrogen ratio (C/N), AHN, and NO_3_^–^-N all significantly increased (*P* < 0.05), with NO_3_^–^-N showing a particularly pronounced increase (*P* < 0.0001). Compared to the N0, EC in N1 and N2 increased by 22.83% and 46.21%, respectively. NO_3_^–^-N content rose by 48.75 mg/kg and 98.40 mg/kg, respectively. The C/N ratio was significantly higher in N1 than in N0. Compared to N0, NH_4_^–^-N content increased by 3.87 mg/kg and 2.32 mg/kg in the N1 and N2 treatments, respectively, but only the difference between N1 and N0 reached statistical significance. TN, TC, OM, and AP levels showed an upward trend as nitrogen concentration increased while AK levels initially rose and then declined; however, none of these changes reached statistical significance. In summary, nitrogen fertilizer addition must be maintained within an appropriate range. Moderate addition can enhance soil nutrient content, while excessive addition exacerbates soil acidification.

**FIGURE 1:**
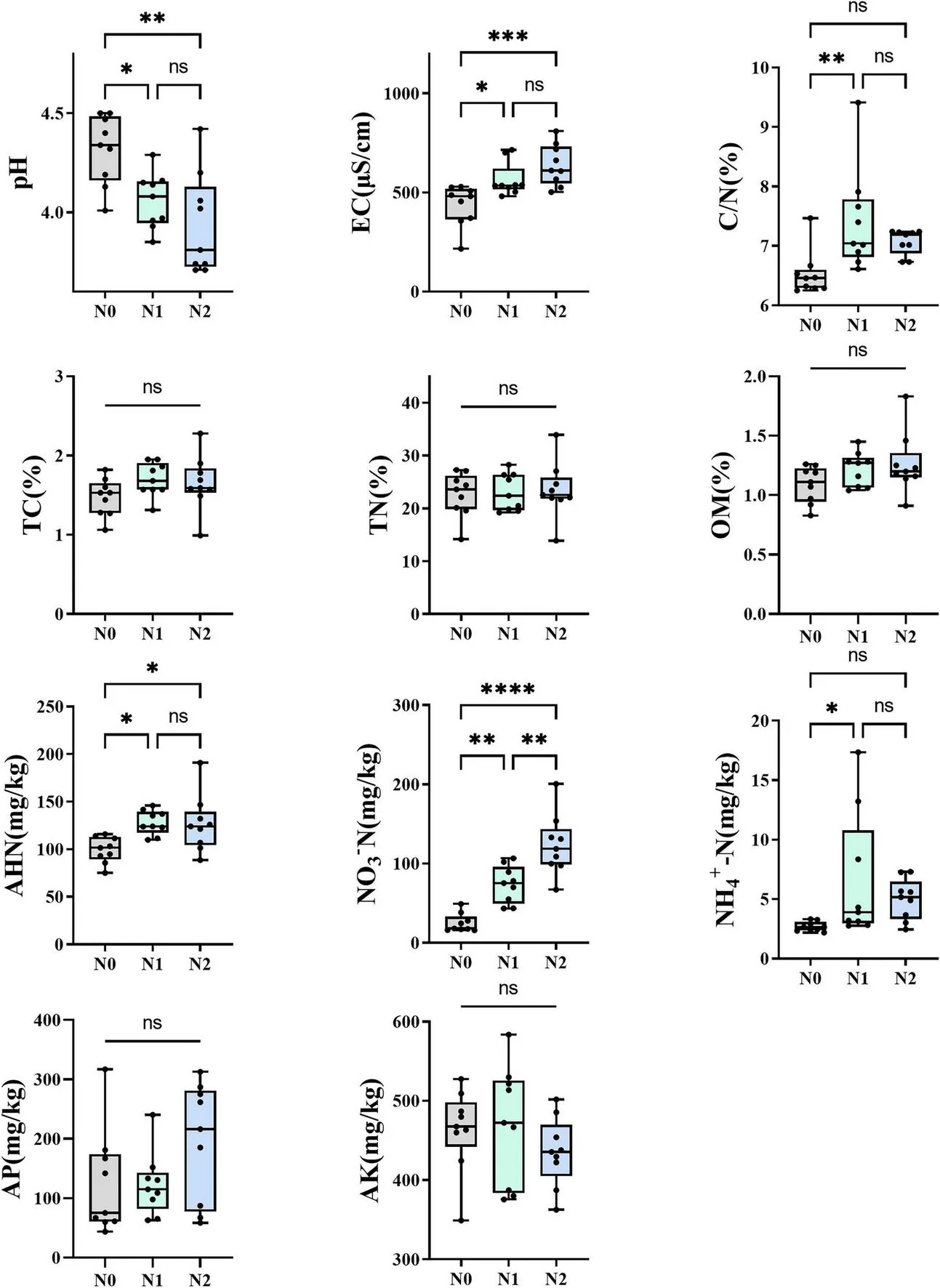
Soil physicochemical responses to nitrogen addition gradients (*n =* 9). ns, not significant; *significant at *P* < 0.05; **P < 0.01; ***P < 0.001; ****P < 0.0001.

### 3.2 Nitrogen cycling gene abundance

In this study, urea was applied to the soil as an external nitrogen fertilizer. Through urease hydrolysis, it was converted into NH_4_^–^, subsequently participating in the soil nitrogen cycle (Figure 2A). With increasing nitrogen concentration, the overall abundance of microbial functional genes involved in nitrification (AOA-*amoA*, AOB-*amoA*), ammonification-related genes (*ureC*), and key denitrification genes (*narG*, *nirK*, *nirS*, *nosZ*) exhibited a decreasing trend (Figure 2B). Nitrogen addition significantly reduced the abundance of most nitrogen cycling genes, with particularly pronounced decreases in the abundance of AOA-*amoA*, *ureC*, *narG*, *nosZ*, and *nirK* genes (*P* < 0.05). Specifically, the abundance of the nitrifying gene AOA-*amoA* was significantly higher in the N0 than in the N2, and also significantly higher in the N1 than in the N2. The abundance of the ammonification gene *ureC* was significantly higher in the N0 than in both the N1 and N2, but no significant difference was observed between the N1 and N2 treatments. Among denitrification genes, the abundances of *narG*, *nirK*, and *nosZ* showed significant differences between N0 and N2 treatments. However, the abundances of AOB-*amoA* and *nirS* genes exhibited weaker responses to nitrogen addition and did not show a significant decreasing trend. In summary, nitrogen addition reduced the abundance of genes associated with nitrification, ammonification, and denitrification in soil, but different genes exhibited varying sensitivities to nitrogen addition.

**FIGURE 2:**
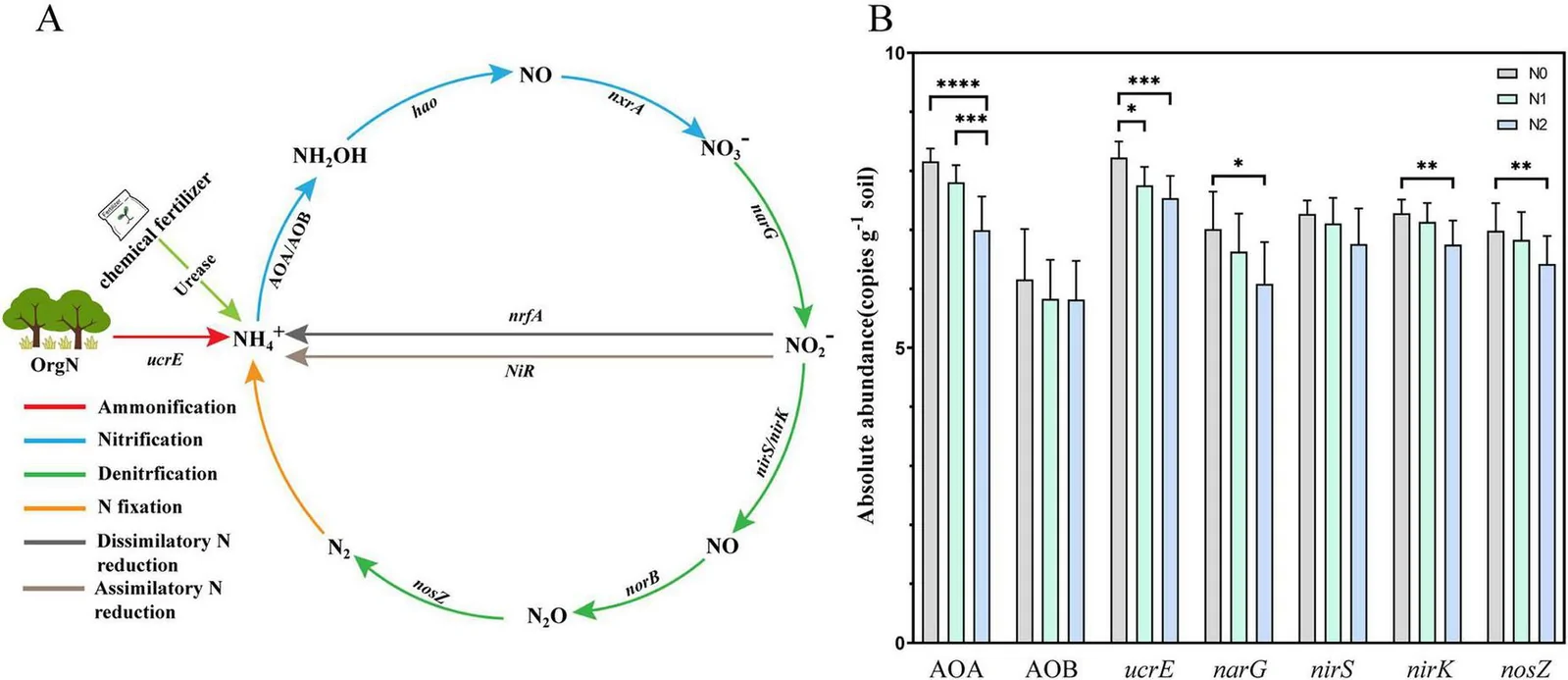
Nitrogen-cycling pathway **(A)** and abundance of involved nitrogen cycling genes **(B)**. AOA and AOB represent AOA-*amoA* and AOB-*amoA* gene abundance, respectively. Data in **(B)** are means ± SE. Significance markers in **(B)**: *P < 0.05; **P < 0.01; ***P < 0.001; ****P < 0.0001 (*n =* 9).

### 3.3 Soil enzyme activity

To compare the effects of different nitrogen treatments on the enzymatic activities of AMO, NIR, NAR, and UE, intergroup statistical tests were performed for each indicator. No significant differences in the activities of these four enzymes were detected across the three nitrogen addition treatments (Figure 3). Although statistical significance was lacking, some directional trends could be observed. With increasing nitrogen addition, AMO activity tended to decrease. NAR activity exhibited a rise-then-fall pattern; NIR activity decreased initially and subsequently recovered; UE activity showed a decrease followed by an increase.

**FIGURE 3:**
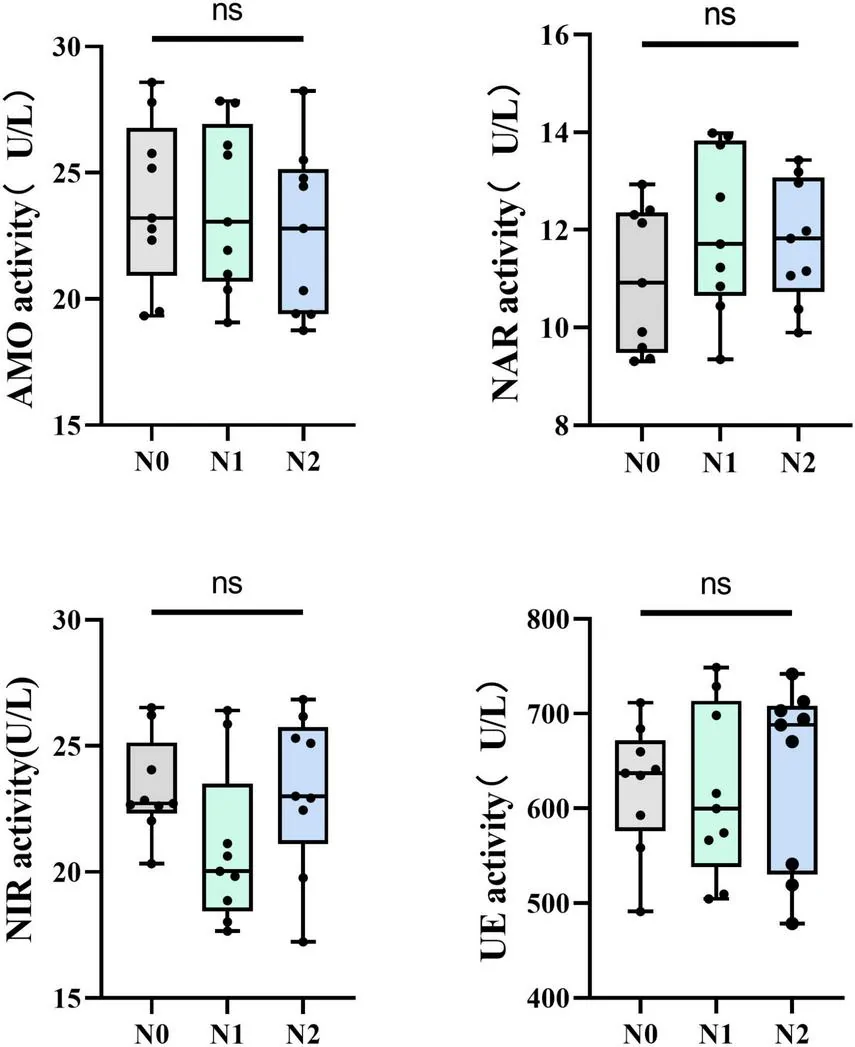
Effects of nitrogen addition on the activities of ammonia monooxygenase (AMO), nitrate reductase (NAR), nitrite reductase (NIR), and urease (UE) (*n =* 9). ns, not significant.

### 3.4 Effects of nitrogen addition on macadamia soil bacteria

Analysis of microbial α-diversity revealed significant differences among the different nitrogen addition treatments. As nitrogen concentration increased, the Chao1, Shannon, and Simpson (Figures 4A–C) indices all decreased significantly (*P* < 0.05), indicating that nitrogen addition significantly reduced both species richness and evenness of the microbial community, leading to an overall decline in α-diversity. β-Diversity analysis further revealed structural differentiation within the communities. PCoA revealed a clear differentiation trend among the microbial communities of the three treatments, and PERMANOVA confirmed significant differences in microbial community composition across treatments (pseudo-F = 4.419, *R^2^* = 0.269, *P* = 0.003; Figure 4D). The multivariate dispersion test was non-significant (F = 0.097, *P* = 0.919), indicating homogeneous within-group dispersion and excluding the possibility that the community difference originated from unequal group dispersion. Consistent results were obtained from UPGMA clustering based on Bray-Curtis distances (Figure 4E), with N0 and N1 clustering together and N2 forming a distinct branch, demonstrating that high-level nitrogen addition significantly altered soil microbial community composition.

**FIGURE 4:**
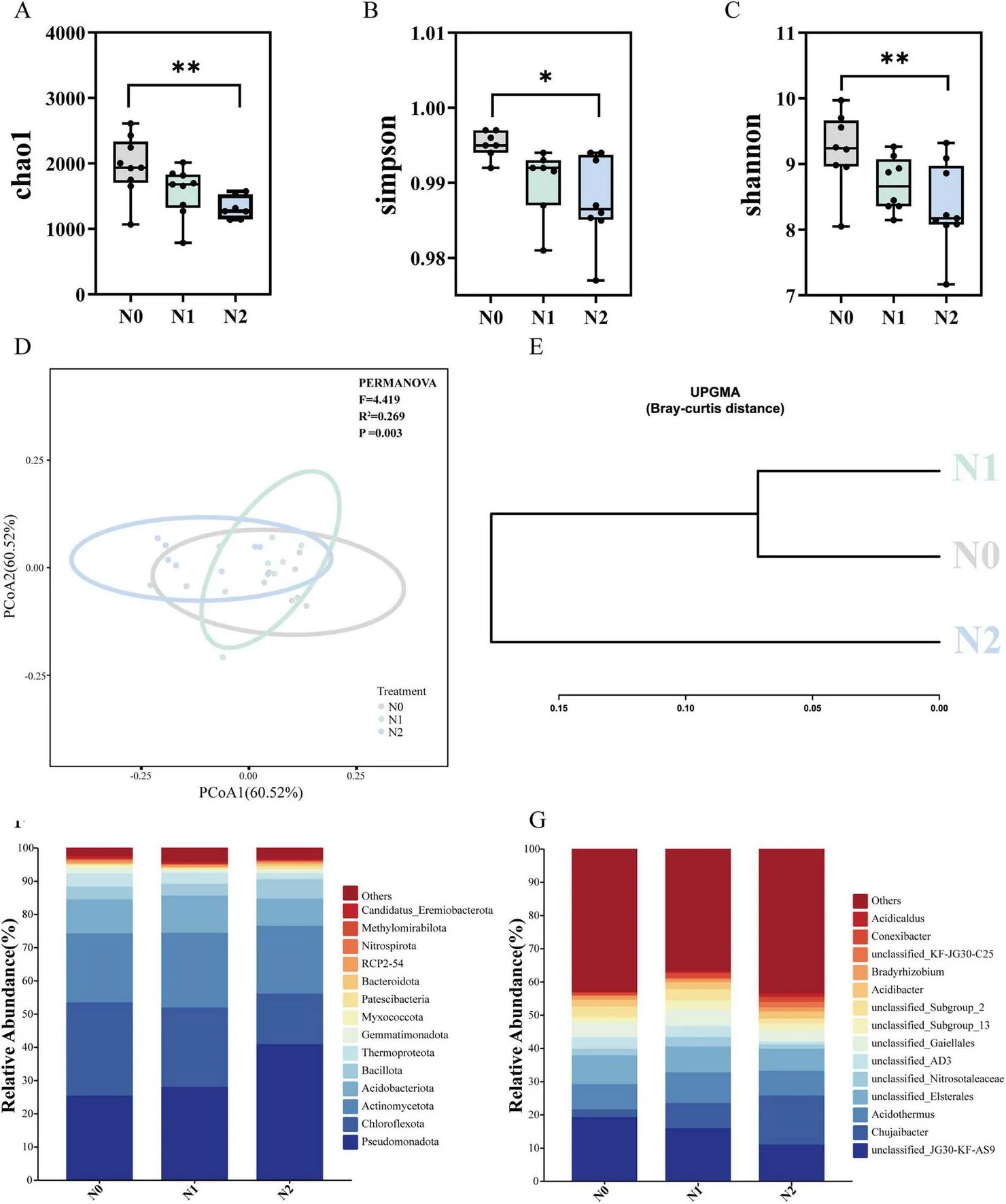
The impact of nitrogen addition on the α-diversity of soil microbial communities an evaluation based on Chao1 **(A)**, Shannon **(B)**, and Simpson **(C)** indices (*n =* 9). *significant at *P* < 0.05; **P < 0.01. The impact of nitrogen addition on the β diversity of soil microbial communities: PCoA **(C)** and UPGMA cluster analysis **(E)** based on Bray-Curti’s distance. PERMANOVA, pseudo-F = 4.419, *R*^2^ = 0.269, *P* = 0.003; homogeneity-of-dispersion test, F = 0.097, *P* = 0.919. Soil microbial community composition under different nitrogen levels showing the 15 most abundant phyla **(F)** and genera **(G)**.

At the phylum level (Figure 4F), nitrogen addition significantly affected microbial community composition. Across the three treatments in macadamia orchard soils, a total of 46 bacterial phyla and 764 bacterial genera were identified. For further analysis, the top 15 most abundant phyla and genera were selected. Pseudomonadota (25.52%, 27.96%, 40.41%) was the most dominant bacteria in all treatments, and its relative abundance increased significantly with the increase of nitrogen concentration (*P* < 0.001). On the contrary, the abundance of chloroflexota (27.70%, 23.57%, 15.47%) decreased significantly with nitrogen addition (*P* < 0.05). Actinomycetota (20.69%, 22.30%, 20.36%) and Acidobacteriota (10.11%, 11.00%, 8.17%) increased first and then decreased. In addition, the abundance of Gemmatimonadota (1.94%, 0.88%, 0.84%) and Bacteroidota (0.17%, 0.18%, 0.70%) decreased, while that of Patencibacteria (0.38%, 0.36%, 0.87%) increased significantly (*P* < 0.05). Other phyla, such as Nitrospirota, Methylmirabilota, Myxococota, Bacillot, Thermoproteota and Candida Eremiobacterota, also declined to varying degrees.

At the genus level (Figure 4G), with the increase of nitrogen concentration, the dominant genera *unclassified_jg30-kf-as9* (19.22%, 15.73%, 11.33%) gradually decreased. *Acidothermus* (7.60%, 9.18%, 7.41%) increased first and then decreased, *Chujaibacter* (2.24%, 7.77%, 14.77%) increased significantly (*P* < 0.0001). At the same time, *unclassified_AD3* (3.41%, 3.48%, 0.92%), *unclassified_Subgroup_2* (3.21%, 0.37%, 1.47%) And *unclassified kf. jg30-c25* (0.28%, 0.34%, 1.65%) decreased significantly (*P* < 0.05), while *Conexibacter* (0.78%, 1.55%,1.62%) and *Acidicaldus* (0.27%, 0.59%, 1.03%) increased significantly (*P* < 0.01).

### 3.5 Soil microbial co-occurrence network

Genus-level bacterial co-occurrence networks were individually constructed for each nitrogen treatment (*n =* 9). Zero-abundance values were retained without imputation. Genera with prevalence < 15% or mean relative abundance < 0.00003 were excluded. Pairwise Spearman correlations were calculated and adjusted via FDR correction. Only correlations with FDR-adjusted *P* < 0.05 and | *r*| > 0.5 were retained, and nodes with degree < 2 were removed (Liu et al., 2021). Taxa were filtered independently per treatment, yielding 413, 410 and 374 valid genera for N0, N1 and N2 networks, respectively. Network robustness was verified by sensitivity analysis using three correlation thresholds (| *r*| > 0.45, 0.50, 0.55) without degree-based node pruning, and degree-preserving null-model permutations (1000 iterations). A modularity *SES* > 1.96 indicated significant non-random network structure. Network edges represent statistical correlations rather than direct biological interactions (e.g., cooperation or competition).

The number of nodes, the number of edges, and the average degree all increase from N0 to N1, but decrease under N2. Compared to N0, node count in N1 increased by 37.69%, and edge count increased by 44.15%. In contrast, node count in N2 decreased by 35.19%, and edge count decreased by 72.18%. Modularity exhibited an increasing trend with rising nitrogen concentration. This trend suggests that, as nitrogen concentrations increase, the network structure of the soil microbial community tends to become more modular, with more pronounced compartmentalization.

### 3.6 Functional spectrum analysis of microbial community based on KEGG pathway

To investigate differences in microbial community function among different treatments, this study utilized Tax4Fun to predict functional profiles based on 16S rRNA gene sequences. At the KEGG Level-1, nitrogen addition significantly altered the functional structure of the microbial community. Specifically, the abundance of Cellular Processes followed a trend of N2 > N0 > N1 (*P* < 0.01), while the abundance of Metabolism showed a trend of N1 > N0 > N2 (*P* < 0.01). Further analysis at the Level-2 revealed that the increase in Cellular Processes was primarily driven by Cellular community – prokaryotes and Cell motility (*P* < 0.05), both of which reached their highest levels in the N2 treatment; the decrease in Metabolism was mainly reflected in the N1 > N0 > N2 trend observed in Carbohydrate metabolism and Biosynthesis of other secondary metabolites (*P* < 0.05). At the L3 level, Quorum sensing and Ribosome biogenesis were significantly higher predicted relative abundance in the N2 treatment (*P* < 0.05), while ABC transporters, Histidine metabolism, and Tyrosine metabolism were significantly lower predicted functional potential under N2 treatment (*P* < 0.05), further corroborating the changes in the macro-functional profile.

### 3.7 Correlations between environmental factors, microbial communities, and nitrogen cycling genes

We used RDA to elucidate the relationships among soil environmental factors, nitrogen cycling genes, and microbial community structure. The results demonstrated that the selected environmental factors explained a substantial proportion of the variation in nitrogen cycling genes. At the gene level, the first two RDA axes accounted for 91.42% of the total constrained variation (RDA1: 77.61%; RDA2: 13.81%), and the model was statistically significant (*P* = 0.0001, adjusted *R^2^* = 0.61). At the bacterial community level, these axes explained 96.31% of the total constrained variation (RDA1: 86.45%; RDA2: 9.86%); however, the model was not statistically significant (*P* = 0.441, adjusted *R*^2^ = 0.036) (Figures 5A, B). This indicates that the measured environmental factors have a stronger and more direct impact on nitrogen cycling genes, without significantly shaping the composition of the overall bacterial community. To further investigate the relationship between environmental factors, nitrogen cycling genes, and bacterial phyla, we conducted correlation analysis using Mantel test. Mantel results indicated that pH, EC, and NO_3_^–^-N were the important environmental factors affecting the functional gene groups involved in nitrification, ammonification and denitrification within the nitrogen cycle (*P* < 0.05; Figure 5C). Significant correlations were also found between several environmental variables and individual bacterial phyla. At the community level, distinct environmental factors were significantly correlated with specific bacterial phyla: *Bacillota* showed strong positive correlations with AK and TN (*P* < 0.01); NO_3_^–^-N was significantly positively correlated with *Chloroflexota*, *Cyanobacteriota*, and *Pseudomonadota* (*P* < 0.01). *Pseudomonadota* also exhibited significant positive correlations with AHN and TC, and *Nitrospirota* was positively correlated with AMO (Figure 5D). Mantel test analyses further revealed significant associations between key nitrogen cycling genes and specific bacterial phyla, indicating that distinct microbial taxa govern different nitrogen transformation processes. Specifically, AOA were significantly positively correlated with *Chloroflexota* and *Pseudomonadota*, whereas AOB showed a significant positive correlation with *Nitrospirota*. Additionally, positive correlations were observed between *ureC* and *Bacillota*, as well as between *nosZ* and *Actinomycetota* (Figure 5E).

**FIGURE 5:**
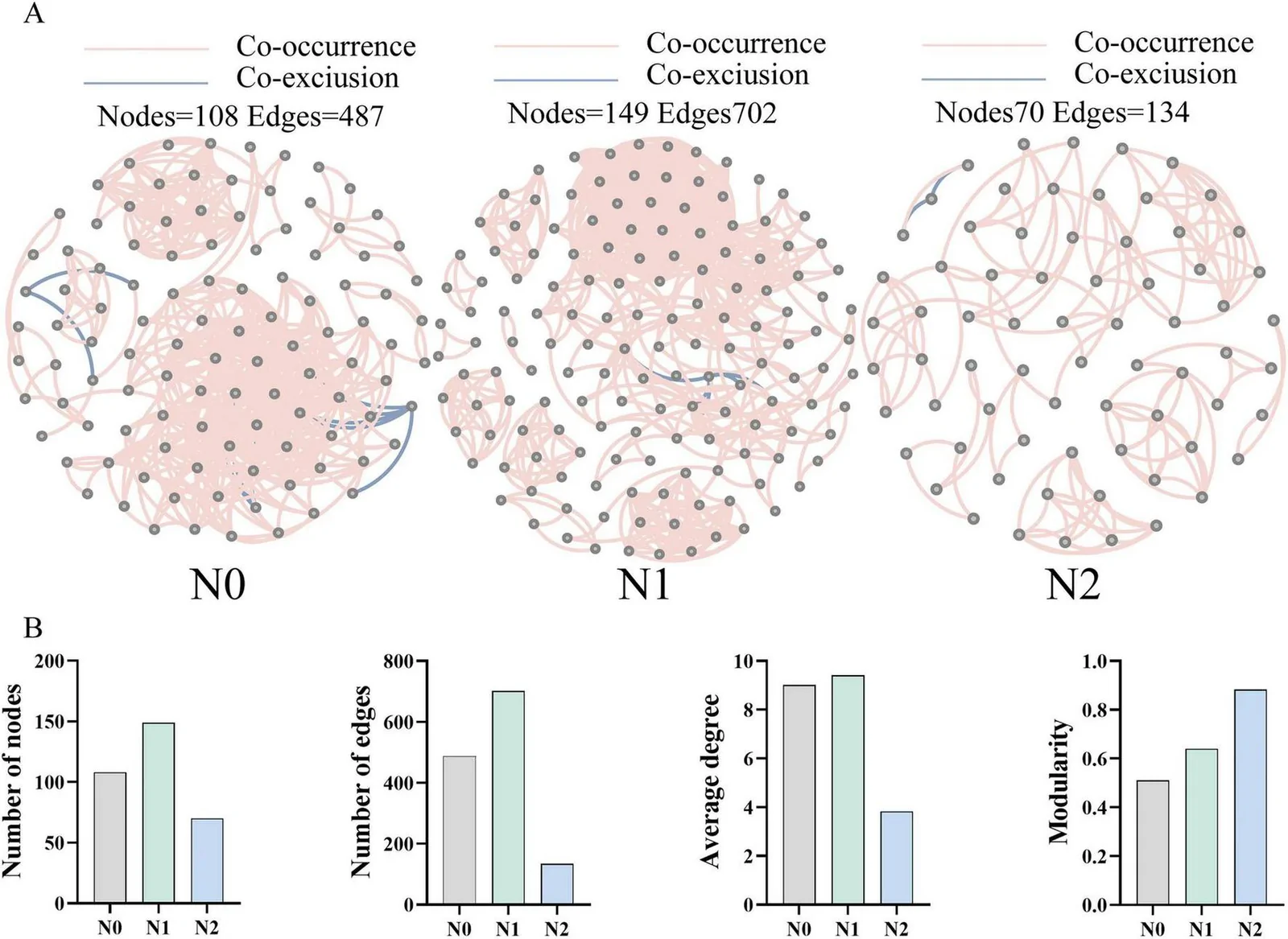
RDA between nitrogen genes and environmental factors **(A)**. RDA between bacterial community composition and environmental factors **(B)**. Relationship between nitrogen cycling genes and environmental factors **(C)**. Relationship between bacterial community composition and environmental factors under different nitrogen levels **(D)**. Relationship between nitrogen-cycling genes and bacterial community composition **(E)** (*n =* 9).

## 4 Discussion

### 4.1 Nitrogen addition alters soil chemical properties

This study revealed that nitrogen addition significantly altered soil chemical properties. Specifically, nitrogen addition increased soil available nitrogen content, including AHN and NO_3_^–^-N (Figure 1), while significantly modifying soil pH and EC. In this study, nitrogen addition significantly altered the stoichiometric relationships and markedly increased the soil C/N ratio (Figure 1). The effects of nitrogen addition on soil stoichiometric balance varied significantly across different ecosystems. In grassland ecosystems, nitrogen addition typically reduces the soil C/N ratio or leaves it unchanged (Meng et al., 2026). However, similar increases in the C/N ratio have also been observed in other ecosystems. For example, in nitrogen-limited desert moss ecosystems, the addition of small amounts of nitrogen can cause the C/N ratio to rise in the short term (Liu et al., 2020). The response of soil stoichiometry to nitrogen addition varies depending on ecosystem type and the duration of fertilization (Han et al., 2019; Buthelezi and Buthelezi-Dube, 2025). Under the N1 treatment, plants may allocate more photosynthetic carbon to the root system, thereby increasing root carbon input and enhancing root growth and exudation. The labile compounds in root exudates promote soil organic carbon accumulation through direct adsorption and indirect stimulation of microbial growth and necromass production (Cao et al., 2025). At the same time, since the study subjects were 10-year-old macadamia trees in their peak fruiting and vegetative growth phases, the added nitrogen was extensively absorbed and utilized by the plants, resulting in a limited net increase in soil TN (Jiang et al., 2024). The functional morphological prediction results (Figure 6B) showed that the carbohydrate metabolism pathway was significantly higher predicted relative abundance under the N1 treatment, confirming this.

**FIGURE 6:**
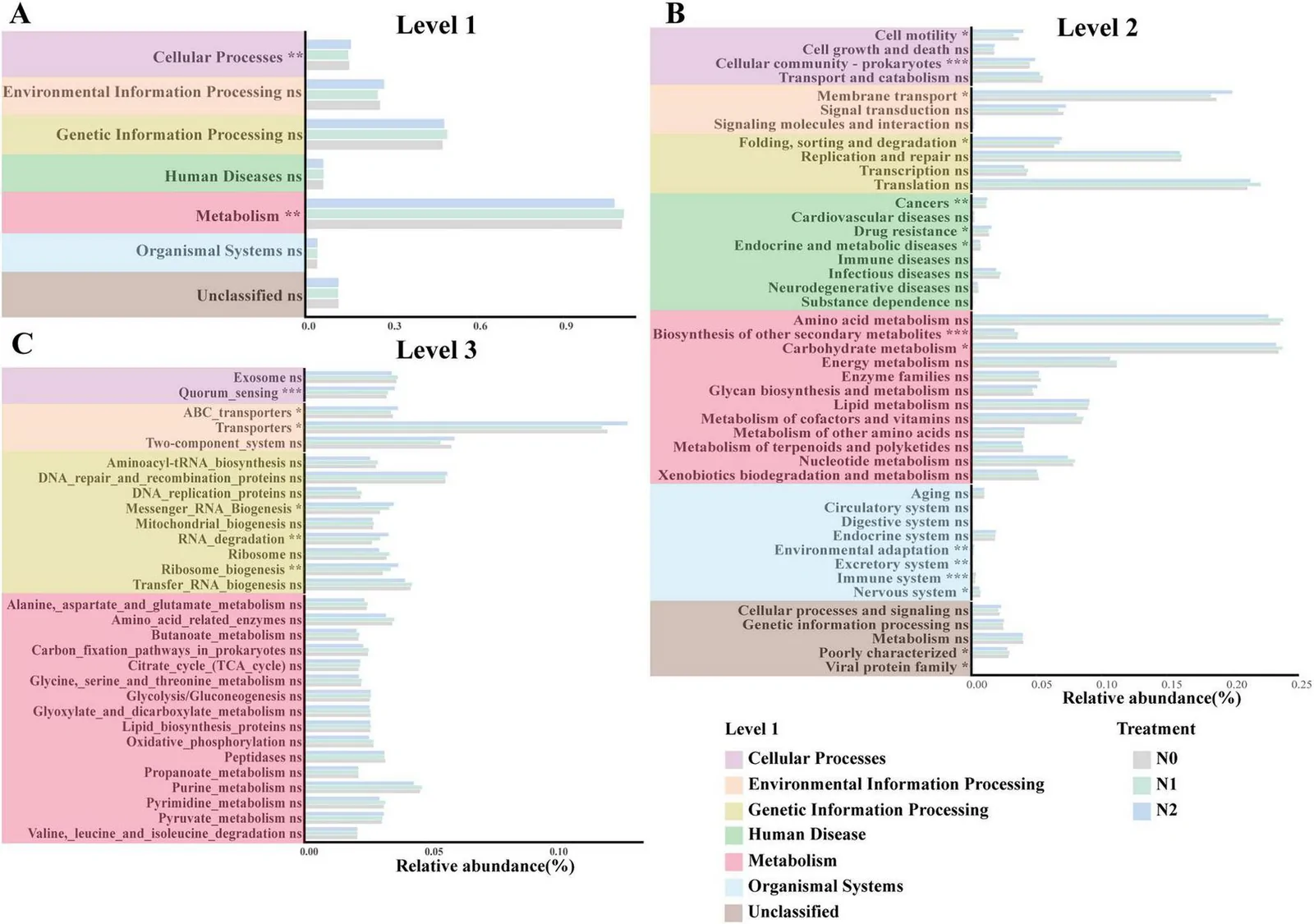
Relative abundances of KEGG Level 1, 2, and 3 pathways obtained using Tax4Fun functional prediction. **(A)** Relative abundances of Level-1 pathways, with different colors representing different pathways; **(B)** Relative abundances of Level-2 pathways; **(C)** The top 30 most abundant Level-3 pathways. One-way ANOVA was used to test for differences in the abundance of pathways at each level across the three treatments. ns, not significant; *significant at *P* < 0.05; **P < 0.01; ***P < 0.001; (*n =* 9).

### 4.2 Differential effects of nitrogen addition on enzyme activities and gene abundances related to nitrogen cycling

The results of this study indicate that nitrogen addition significantly reduced the abundances of both nitrification and denitrification genes. These results are consistent with findings from a meta-analysis of denitrification processes in Chinese ecosystems, which also demonstrated that nitrogen addition significantly reduced the abundance of *narG*, *nirS*, *nirK*, and *nosZ* (Figure 2B) (Dai et al., 2025). Previous studies have shown that as pH decreases, AOB generally declines, while AOA tends to increase in most cases. However, extremely low pH (close to 5.0) has an inhibitory effect on AOA (Ying et al., 2017). In this study, the soil pH was below 5.0 (Figure 1), representing an extremely acidic environment, which may be the primary cause of the reduced abundance of AOA-*amoA* (Figure 2B). Furthermore, previous studies have shown that the copy number of the *ureC* gene is significantly correlated with increasing pH, meaning that *ureC* abundance increases as pH rises (Fisher et al., 2017). In this study, the abundance of *ureC* significantly decreased (Figure 2B), which may be related to a decrease in soil pH. Results from RDA and the Mantel test further indicate that pH, EC, and NO_3_^–^-N are important environmental factors affecting the composition of nitrogen cycling gene groups (Figure 5C).

In this study, we observed an asynchronous trend between the activities of key nitrogen metabolizing enzymes and the abundances of their encoding genes. For instance, the abundance of the *amoA* gene, which encodes the AMO enzyme (Araki et al., 2004), decreased significantly with increasing nitrogen concentration. However, AMO activity showed no significant differences among treatments. This pattern suggests potential functional redundancy (Allison and Martiny, 2008; Schauss et al., 2009). In contrast, the activities of NAR, NIR, and UE exhibited asynchronous patterns relative to gene abundances. Specifically, NAR activity increased with substrate (NO_3_^–^) (Ingle, 1966) availability under the N1 treatment but decreased under the N2 treatment despite further increases in NO_3_^–^-N, potentially attributable to competition for electron donors (e.g., NADH) (Wang and Qiao, 2023) or enzyme activity saturation effects. Regarding NIR, activity decreased in the N1 treatment, likely due to substrate inhibition by NO_2_^–^ generated via NAR catalysis (Neubauer and Götz, 1996). The subsequent recovery, however, may be driven by differences in ecological adaptation between *nirS*- and *nirK*-type denitrifying communities (Ming et al., 2024). These two community groups displayed markedly different responses to nitrogen addition, and the enrichment of specific subpopulations under high-nitrogen conditions may promote NIR activity (Pold et al., 2024). Regarding the relationship between urease activity and the abundance of its encoding gene *ureC*, previous studies have reported inconsistent conclusions. Sun et al. observed a concurrent stimulation of both urease activity and *ureC* abundance by nitrogen addition, although this relationship may be modulated by factors such as soil depth (Sun et al., 2019). Wang L. et al. (2018) and Ouyang and Norton (2020) found no correlation between urease activity and *ureC* gene copy number in fertilization experiments in northeastern black soil and in composting experiments, respectively, indicating that changes in the abundance of *ureC* do not directly reflect alterations in urease function. Our results support this latter view. In the present study, urease activity was also not significantly correlated with *ureC* gene copy numbers, further indicating that changes in *ureC* abundance do not directly correspond to alterations in urease function.

In summary, the differences among various nitrogen-metabolizing enzymes in post-transcriptional regulation, functional redundancy, substrate limitation, and responses to environmental factors suggest that relying solely on functional gene abundance cannot accurately predict enzyme activity levels. Combining enzyme activity assays with gene abundance analyses is therefore essential for understanding the regulatory mechanisms of the nitrogen cycle.

### 4.3 Characteristics of nitrogen addition responses in microbial community structure

In this study, nitrogen addition altered the structural composition of soil microbial communities. Cluster analysis based on community composition revealed that N0 and N1 clustered together, while N2 branched independently (Figure 4D). This result indicates that the overall structure of soil microbial communities in the N1 treatment remained relatively stable; in the N2 treatment, the community structure underwent significant differentiation. Analysis at the phylum level further revealed (Figure 4F) that nitrogen addition altered shifts in microbial ecological strategies, manifested as the expansion of copiotrophic (R-strategy) microbial taxa and the decline of oligotrophic (K-strategy) microbial taxa. Specifically, the relative abundance of the copiotrophic representative phylum *Pseudomonadota* increased significantly with increasing nitrogen concentration, whereas the relative abundance of the oligotrophic representative phylum *Chloroflexi* decreased significantly, a pattern consistent with findings from a study on alpine meadows on the Qinghai–Tibet Plateau (Zhao et al., 2025). Meanwhile, *Actinobacteriota* and *Acidobacteriota* exhibited an initial increase followed by a decrease, suggesting a degree of tolerance to medium nitrogen levels, with high-nitrogen conditions suppressing their growth. Furthermore, the decline in the abundances of several other phyla further corroborated the selective effect of nitrogen addition on sensitive taxa.

Changes at the genus level (Figure 4G) were highly consistent with those at the phylum level. Among R-strategy (copiotrophic) taxa, *Acidicaldus* (phylum *Pseudomonadota*), *Chujaiobacter* and *Conexibacter* (phylum *Actinobacteriota*) were significantly enriched. Notably, the enrichment of *Acidicaldus*, an acidophilic genus (Liu et al., 2022), may be associated with soil acidification induced by nitrogen addition. In contrast, K-strategy (oligotrophic) taxa exhibited a declining trend, including *unclassified_jg30-kf-as9* and *unclassified_kf.jg30.c25* (phylum *Chloroflexi*), as well as *Acidothermus* and *unclassified_Subgroup_2* (phylum *Acidobacteriota*) (Kalam et al., 2020), reflecting the weakened competitive advantage of oligotrophic taxa under nitrogen addition. Overall, these results indicate that nitrogen addition alters the succession of microbial community structure, steering it toward a eutrophic community succession.

Specific environmental factors are significantly associated with functional genes and bacterial phyla (Figure 5D). The *Nitrospirota* shows a positive correlation with AMO because *Nitrospirota* can gradually oxidize ammonia into nitrate and contains AMO (Kuypers et al., 2018). The Mantel test further revealed significant associations between key nitrogen cycling genes and specific bacterial phyla, indicating that different microbial dominate distinct nitrogen transformation processes (Figure 5E). The *Pseudomonadota* is involved in multiple nitrogen transformation pathways, including nitrification (Sanow et al., 2023), denitrification (Jéglot et al., 2021), ammonia assimilation (Chi et al., 2024), and assimilatory nitrate reduction (Sparacino-Watkins et al., 2014). The *Pseudomonadota* showed a positive correlation with AOA-*amoA* and NO_3_^–^-N, which may reflect the *Pseudomonadotas*’ utilization of NO_3_^–^ produced by AOA mediated denitrification through denitrification or nitrate-reducing pathways (Qin et al., 2013). In this study, a significant positive correlation was observed between the abundance of AOA-*amoA* and that of *Nitrospira*, consistent with the observations of Stempfhuber et al. in an unfertilized grassland ecosystem (Stempfhuber et al., 2016). That study found that AOA and *Nitrospira* were positively correlated at the same spatial scale, leading to the conclusion that these two microorganisms jointly alter the nitrification process. The correlations observed in our study further support this conclusion. *ureC* is positively correlated with *Bacillota*, which belongs to the *ureC* microbial subcommunities (Feng et al., 2025). The *ureC* gene is generally considered to be representative of soil urease activity (Reed, 2001). Therefore, *Bacillota* plays an important role in the urea hydrolysis stage of the ammonification process. The positive correlation between the *nosZ* gene and *Actinomycetota*. Previous studies have shown that *Actinomycetota* carry the *nosZ* gene and participate in the denitrification process (Green et al., 2010).

Nitrogen addition also significantly altered the predicted functional structure of the microbial community (Figure 6). In this study, medium-nitrogen addition was associated with higher predicted relative abundances of functions related to carbohydrate metabolism and the biosynthesis of other secondary metabolites. This response pattern is consistent with previous findings indicating that moderate nitrogen addition enhances soil microbial carbon metabolic activity, while excessive nitrogen addition inhibits this activity (Wu et al., 2017; Weng et al., 2022; Gao et al., 2023). In nitrogen-poor soils, moderate nitrogen addition alleviates nitrogen limitation and increases soil available nitrogen content, thereby enhancing microbial carbon metabolism functions (Liu et al., 2010). Conversely, excessive nitrogen addition may inhibit microbial metabolic activity by inducing soil acidification (Meng et al., 2026). This study also observed that the pH in the N2 treatment was significantly lower than that in the N0 treatment (*P* < 0.01), concurrent with lower predicted carbohydrate microbial metabolic potentials. To further verify whether the reduction in the predicted abundance of carbohydrate metabolism was associated with soil acidification, partial correlation analysis was performed using SPSS 26.0 with nitrogen addition level controlled. After excluding the confounding effect of nitrogen treatment, no significant independent correlation was detected between soil pH and the predicted carbohydrate metabolic abundance (partial r = 0.013, *P* = 0.950). These results suggest that the decline in carbohydrate metabolic potential under high nitrogen may not be primarily explained by soil pH reduction, despite the significant soil acidification induced by high-nitrogen addition. Network analysis indicates that the medium-nitrogen treatment promoted an increase in the number of network nodes and the average degree to some extent, slightly enhancing the network’s connectivity and complexity. By contrast the high-nitrogen treatment reduced the connectivity and complexity of the microbial network (Figure 7). It is worth noting that modularity showed an upward trend; this increase in modularity indicates that microbial communities tend to form functionally more independent sub-networks, which are more tightly connected internally. This implies the formation of a more differentiated network structure, enhancing the system’s resilience to environmental perturbations (Fang et al., 2026). It should be noted that Tax4Fun only infers microbial functional potential from 16 S rRNA gene taxonomic profiles, rather than measuring actual gene expression or in-situ metabolic activity. Thus, functional profiles represent predicted relative abundances rather than real physiological performances.

**FIGURE 7:**
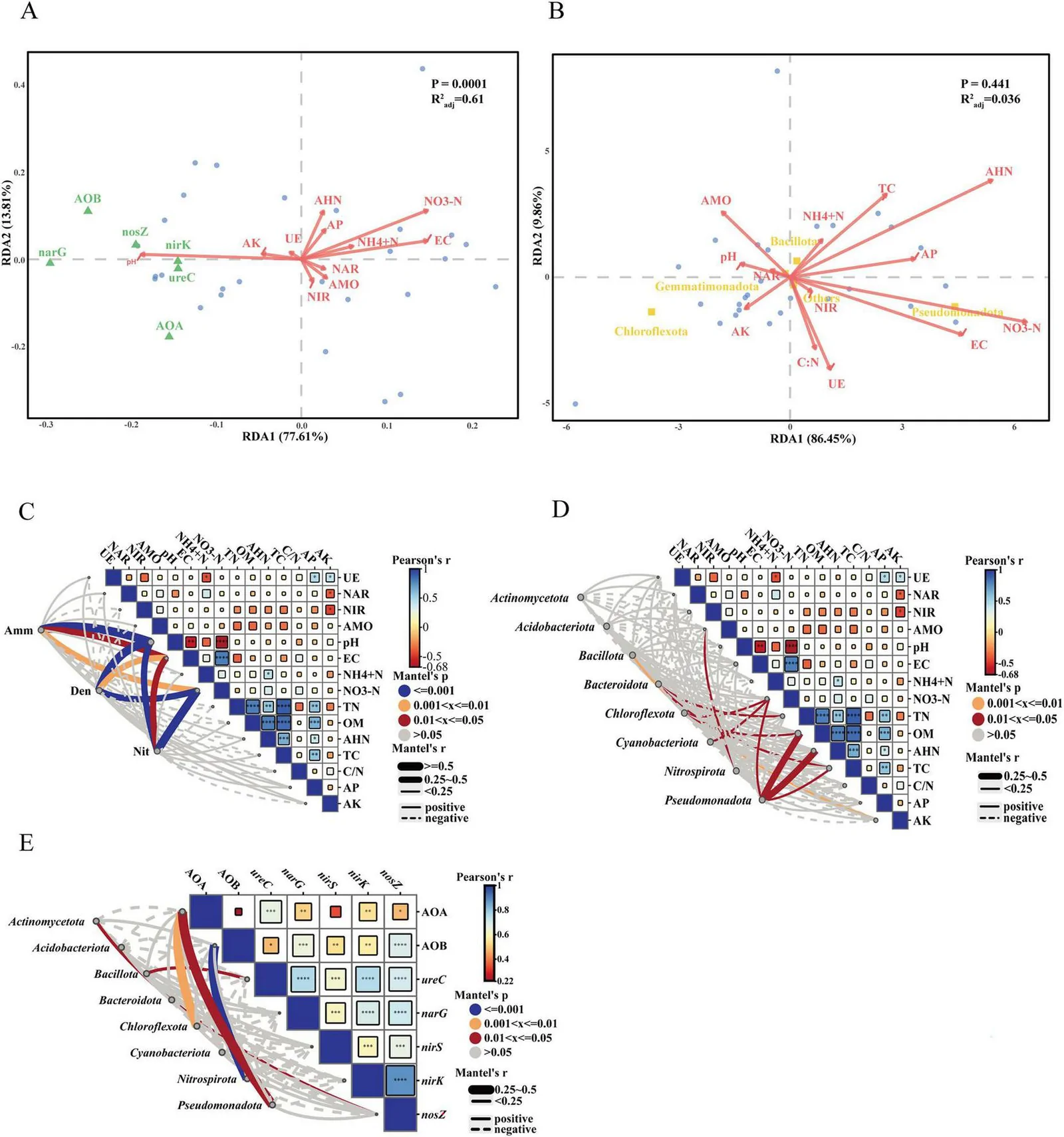
Genus-level distribution of the soil bacterial community based on the co-occurrence network **(A)**. Characterization of network topological properties **(B)**. Pink and blue edges indicate cooperative and competitive relationships between species, respectively (*n =* 9).

## Conclusion

The results of this study indicate that nitrogen addition significantly reduced the abundance of nitrogen cycling genes, restructured the microbial community, and decreased microbial diversity, thereby altering changes in nitrogen cycling processes. Medium-nitrogen maintained relative stability in community structure and corresponded to higher predicted carbon metabolism-related functional potential and network complexity. In contrast, high-nitrogen input altered marked differentiation in community structure, leading to a shift toward a simplified, eutrophic community, accompanied by a lower predicted capacity for microbial metabolic functions and reduced network complexity. Additionally, stable associations exist between key nitrogen cycling genes and specific bacterial phyla, reflecting functional differentiation and synergy among microbial groups during nitrogen transformation. Against the backdrop of intensifying global nitrogen deposition, investigating the characteristics of macadamia soil microbial communities and their response mechanisms to nitrogen addition is of great significance for guiding the sustainable cultivation of this crop.

## Data Availability

The raw 16S rRNA-gene amplicon sequencing data generated in this study have been deposited in the NCBI Sequence Read Archive (SRA) under BioProject accession number PRJNA1517348 (https://www.ncbi.nlm.nih.gov/bioproject/PRJNA1517348). Other datasets supporting the findings of this study are available from the Yunnan Agricultural Engineering Research Center for Macadamia upon reasonable request.
